# HIV-1 gp120 influences the expression of microRNAs in human monocyte-derived dendritic cells via STAT3 activation

**DOI:** 10.1186/s12864-015-1673-3

**Published:** 2015-06-27

**Authors:** Andrea Masotti, Gloria Donninelli, Letizia Da Sacco, Barbara Varano, Manuela Del Cornò, Sandra Gessani

**Affiliations:** Bambino Gesù Children’s Hospital-IRCCS, Viale di San Paolo 15, 00146 Rome, Italy; Department of Hematology, Oncology and Molecular Medicine, Istituto Superiore di Sanità, Viale Regina Elena 299, 00161 Rome, Italy

**Keywords:** microRNA, HIV-1, Dendritic cell, STAT3, gp120

## Abstract

**Background:**

MicroRNAs (miRs) are an abundant class of small non-coding RNAs (~22 nt) that reprogram gene expression by targeting mRNA degradation and translational disruption. An emerging concept implicates miR coupling with transcription factors in myeloid cell development and function, thus contributing to host defense and inflammation. The important role that these molecules play in the pathogenesis of HIV-1 is only now emerging.

**Results:**

We provide evidence that exposure of monocyte-derived dendritic cells (MDDCs) to recombinant HIV-1 R5 gp120, but not to CCR5 natural ligand CCL4, influences the expression of a panel of miRs (i.e., miR-21, miR-155 and miR-181b) regulated by STAT3 and potentially targeting genes belonging to the STAT3 signaling pathway. The blockage of gp120-induced STAT3 activation impairs gp120 capacity to modulate the expression level of above mentioned miRs. Predictive analysis of miR putative targets emphasizes that these miRs share common target genes. Furthermore, gene ontology and pathway enrichment analysis outline that these genes mainly belong to biological processes related to regulation of transcription, in a complex network of interactions involving pathways relevant to HIV-DC interaction.

**Conclusions:**

Overall, these results point to gp120-triggered modulation of miR expression via STAT3 activation as a novel molecular mechanism exploited by HIV-1 to affect DC biology and thus modulate the immune response through complex regulatory loops involving, at the same time, miRs and transcription factors.

**Electronic supplementary material:**

The online version of this article (doi:10.1186/s12864-015-1673-3) contains supplementary material, which is available to authorized users.

## Background

MicroRNAs (miRs) are an abundant class of small non-coding RNAs (~22 nt) that function to control gene expression and restrict viral replication in host cells [1, 2]. Dysregulation of miRs expression is associated with numerous disease states. The human genome encodes hundreds of miRs with the potential to regulate up to 92 % of genes, mostly through inhibition of translation and/or promotion of mRNA degradation. Growing evidence indicates that miRs and transcription factors can jointly regulate target gene expression in the form of feed-forward or feedback loops [3]. These regulatory loops serve as important motifs in functional networks and play critical roles in multiple biological processes, either in healthy states or diseases. miR-mediated gene silencing has been shown to be a key event in viral pathogenesis [4–6]. Several cellular miRs show substantial changes in expression upon HIV-1 infection [6, 7], and contributes to viral latency in primary CD4^+^ T lymphocytes [8]. HIV-1 infectivity has been reported to be influenced by cellular miRs [9]. Multiple cellular miRs can modulate both viral infectivity and replication by targeting directly the viral genome or decreasing the expression of host proteins required for virus replication [1]. More recently, changes in miRs expression profile of CD4^+^ T lymphocytes after exposure to HIV-1 allowed to discriminate among different stages of HIV infection [10].

Dendritic cells (DCs) are professional antigen presenting cells playing a critical role in the orchestration and fine-tuning of the immune response [11]. At the mucosal sites, they are primary targets of HIV-1 infection, co-opted by the virus to facilitate its transfer to T lymphocyte and body dissemination [12]. However, how HIV-1 manipulates DC biology to use these cells to its own advantage without marked cytopathic effects is still unclear. In these cells, the interaction of the envelope protein gp120 with surface receptors triggers early signaling events even in the absence of productive infection and may profoundly influence cellular behavior and secretory profile [13]. In this respect, we have recently reported that DC exposure to HIV-1 R5 gp120 resulted in production of IL-6 via MAPK/NF-kB pathways which, in turn, activated STAT3 by an autocrine loop [14]. This modulation is dependent on C-C chemokine receptor 5 (CCR5), the principal coreceptor of HIV-1 entry in DC, and it is specific for gp120, since the C-C chemokine ligand 4 (CCL4), the most specific natural ligand for CCR5, did not induce IL-6 production and STAT3 activation in these cells [14].

Interestingly, a growing body of evidence demonstrates that miRs are closely associated with the STAT3 signaling pathway supporting the existence of regulatory feedback loops between miRs and several components of the STAT3 pathway in different cancer contexts [15].

In this study, we performed a bioinformatics analysis to identify a panel of miRs that present one or more STAT3 binding sites in their promoter region. Moreover, we selected miR targeting genes belonging to STAT3 signaling pathway and determined whether the addition of gp120 to monocyte- derived DCs (MDDCs) would alter their expression levels. We found that gp120-triggered STAT3 activation directly influenced the expression of miR-21, miR-181b and miR-155. Bioinformatics analysis of miR putative targets unraveled that these genes mainly belong to biological process related to ‘transcription’ in a complex network of interactions relevant to HIV-DC biology. Finally, we validated a selection of putatively regulated proteins and we showed that the expression of gp120-deregulated miRs is in agreement with the abundance of their target proteins (i.e., PIAS3 and STAT1).

## Results

### Promoters of precursor miRs contain several predicted STAT3 binding sites

We have recently reported that exposure of MDDCs to R5 gp120 activates the STAT3/IL-6 axis [14]. STAT3 has been predicted to regulate the transcription of a great number of miRs and putative STAT3 binding sites were identified in many miR promoters [16, 17]. Starting from these data, we generated a list of miRs that was then used to predict the number of putative STAT3 binding sites. Table 1 shows the result of this prediction by which the number of putative STAT3 binding sites employing the matrix-derived models (JASPAR CORE and TRANSFAC models) and three different STAT3 binding motifs (MA0144.1, M00225 and M00497) have been obtained. Information on position, strand, score value and statistical significance of the predicted STAT3 binding sites are reported in additional file 1.

**Table 1:** Analysis of the promoter regions of putative STAT3 regulated miRs

	Number of predicted STAT3 binding sites			
	MA0144.1	M00225	M00497	Total
hsa-mir-3124	8	12	5	25
hsa-mir-92b	6	12	4	22
hsa-mir-1205	11	4	6	21
hsa-mir-1206	7	12	1	20
hsa-mir-1256	9	8	2	19
hsa-mir-629	4	11	4	19
hsa-mir-146a	2	5	12	19
hsa-mir-3125	10	7	1	18
hsa-mir-1255a	10	6	2	18
hsa-mir-3142	9	7	2	18
hsa-mir-125b-2	8	8	2	18
hsa-mir-155	8	5	4	17
hsa-mir-30e	8	3	5	16
hsa-mir-645	11	2	3	16
hsa-mir-181b-1	6	3	7	16
hsa-mir-1537	9	4	2	15
hsa-mir-3197	6	7	2	15
hsa-mir-21	7	4	4	15
hsa-mir-30c-1	12	1	2	15
hsa-mir-1825	12	0	2	14
hsa-mir-29a	8	2	3	13
hsa-mir-135b	7	2	4	13
hsa-mir-1207	7	2	3	12
hsa-mir-619	6	2	4	12
hsa-mir-548h-1	4	1	7	12
hsa-mir-646	5	4	3	12
hsa-let-7b	8	3	1	12
hsa-mir-125b-1	3	4	5	12
hsa-mir-181a-2	10	1	1	12
hsa-mir-29b-1	5	2	3	10
hsa-mir-3145	3	5	1	9
hsa-mir-612	5	4	0	9
hsa-mir-3174	4	3	2	9
hsa-mir-630	2	4	3	9
hsa-mir-451	8	1	0	9
hsa-mir-29c	6	1	1	8
hsa-mir-202	5	1	1	7
hsa-mir-148b	2	2	3	7
hsa-mir-181b-2	4	3	0	7
hsa-let-7a-3	5	1	0	6
hsa-mir-548c	0	0	2	2

The number of STAT3 binding site for each sequence motif obtained by LASAGNA search is reported for each selected miR

To select miRs specifically related to STAT3 biological processes and directly implicated in the regulation of STAT3 signaling pathway activated in gp120-exposed MDDCs [14], the same miR list was intersected with the group of validated miRs annotated in the miRWalk database whose targets belong to STAT3 signaling pathway. This analysis led to a restricted list of 14 miRs potentially regulated by STAT3 and targeting genes annotated in the STAT3 signaling pathway (Biocarta) that is shown in Table 2. Detailed information on this analysis is provided in additional file 2. Interestingly, some of the miRs reported in Table 2 (i.e., miR-21, miR-125b, miR-135b, miR-181b and miR-155) were already predicted to be regulated by STAT3 and also experimentally validated [16, 18–21].

**Table 2:** List of genes in the STAT3 signaling pathway targeted by STAT3 regulated miR

miRNA name	Targeted genes in STAT3 signaling pathway
hsa-mir-21	MAPK3, FRAP1, STAT3
hsa-mir-155	STAT3, MAPK3, JAK2, JAK1, FRAP1
hsa-let-7b	STAT3, MAPK3, JAK2
hsa-let-7a-3	STAT3, MAPK3, JAK2
hsa-mir-146a	STAT3, MAPK3
hsa-mir-125b-2	MAPK3, FRAP1, STAT3
hsa-mir-125b-1	MAPK3, FRAP1, STAT3
hsa-mir-29a	FRAP1, MAPK3
hsa-mir-135b	STAT3
hsa-mir-451	JAK2, FRAP1
hsa-mir-30c-1	JAK2
hsa-mir-181a-2	MAPK3
hsa-mir-181b-2	STAT3
hsa-mir-181b-1	STAT3

The table shows the genes present in the STAT3 signaling pathway (Biocarta) that are validated targets of a selection of STAT3 regulated miR

### HIV-1 gp120-induced activation of STAT3 modulates miR expression profiles in MDDCs

**Fig. 1:**
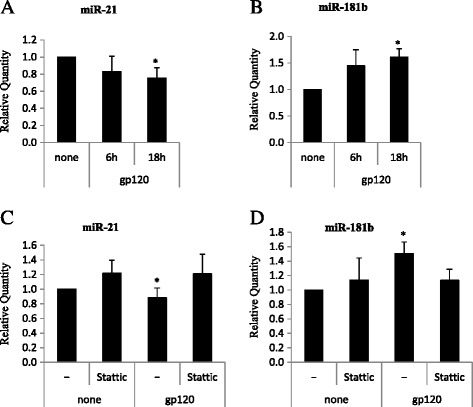
Real-time qPCR of HIV gp120 modulated miRs. Cells were treated with gp120 for 6–18 h or left untreated in the presence or in the absence of Stattic (10 μM, 1 h of pretreatment) and total RNA was extracted, reverse transcribed and subjected to Real-time qPCR. Relative fold of change of expression of miR-21 **a-c**, miR-181b (B-D), in gp120 stimulated MDDCs against untreated controls were calculated using the comparative Ct (2^-ΔΔCt^) method. PCR were run in triplicate and the mean of 12 (A-B) or 5 **c-d** independent experiments ± SE is shown. *p* values were calculated by ANOVA and statistical significance is indicated *vs* untreated control

On the basis of the bioinformatics analyses illustrated above, we first experimentally validated a panel of miRs including miR-21, miR-125b, miR-135b and miR-181b in gp120-treated MDDCs by real-time quantitative PCR (qPCR). As shown in Fig. 1, miR-21 (A) and miR-181b (B) were down- and up-regulated, respectively, in cells treated with R5 gp120 at 18 h. Conversely, no significant difference was observed at 6 h. Likewise, gp120 treatment did not result in any significant modulation of miR-125b, whereas miR-135b did not amplify (data not shown). Although miR modulation was quite modest, it showed reproducibility among all donors tested (n = 12) and results were statistically significant with respect to untreated controls. Then, to better define the role of STAT3 in the gp120-induced modulation of miR-21 and miR-181b, we assessed the expression profile of these miRs in the presence of Stattic, a non-peptidic small molecule inhibiting STAT3 activation and dimerization [22]. Although the addition of Stattic did not *per se* modulate the baseline expression levels of these miRs, it completely abolished the gp120-induced down-modulation of miR-21 (C) as well as the up-modulation of miR-181b (D), thus confirming a role for STAT3 in the regulation of these miRs in MDDCs.

**Fig. 2:**
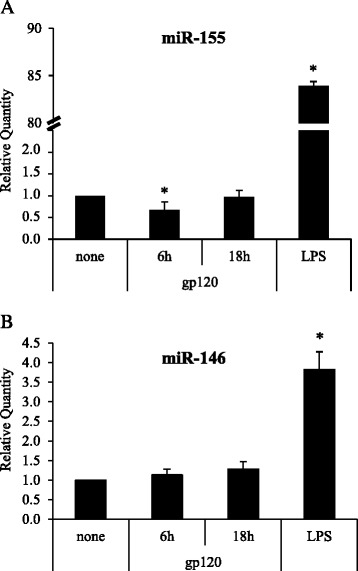
Real-time qPCR of miR-155 and miR-146. Cells were treated, and relative FC of expression of miR-155 **a** and miR-146 **b** in gp120- or LPS- stimulated MDDCs against untreated controls were calculated as described in Fig. 1. PCR were run in triplicate and the mean of 8 independent experiments ± SE is shown. *p* values were calculated by ANOVA and statistical significance is indicated *vs* untreated control

HIV-1 gp120 and LPS have been reported to induce, at some extent, overlapping effects in a variety of cell types, as both molecules promote cytokine/chemokine secretion and modulate cell activation state. Furthermore, MDDCs activated by LPS show a typical miR expression profile that includes a remarkable up-modulation of miR-155 and to a lesser extent, of miR-146 [23–26]. Of note, both miRs have been reported to play an important role in the modulation of the immune response [23, 27]. As shown in Fig. 2, while LPS markedly up-modulated miR-155 and miR-146 expression, at early time points (6 h), gp120 exhibited a significant inhibitory effect (1.4 fold decrease) on miR-155 that disappeared at later time points (18 h), while miR-146 was not affected.

In keeping with our previous observation that CCL4 does not affect the STAT3/IL-6 axis [14], this chemokine, known as the most specific natural ligand engaging CCR5, did not alter the expression of miR-21, miR-155 and miR-181b expression, as well as that of miR-146 (Table 3).

**Table 3:** Effects of CCL4 on miR-21, miR-146, miR-155 and miR-181b

	Control	CCL4 (6 h)	CCL4 (18 h)	*p*-value
miR-21	1	0.80 ± 0.31	0.80 ± 0.36	0.642
miR-146	1	1.10 ± 0.29	1.38 ± 0.23	0.347
miR-155	1	1.15 ± 0.69	1.11 ± 0.06	0.932
miR-181b	1	1.31 ± 0.38	1.51 ± 0.21	0.277

Fold changes and standard errors of miR-21, miR-146, miR-155 and miR-181b expressed in DCs after CCL4 treatment (6 and 18 h). *p* values were calculated by ANOVA and statistical significance is indicated *vs* untreated control (n = 5)

### Gene Ontology of miR-21, miR-155 and miR-181b predicted targets

**Fig. 3:**
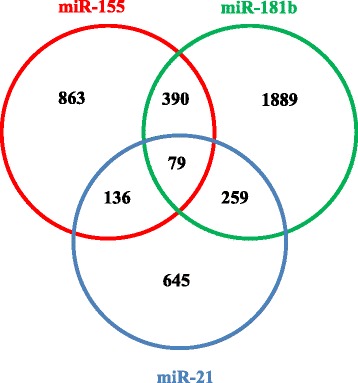
Overlap of predicted genes targeted by STAT3-regulated miRs. Venn diagram showing the overlap between predicted target genes of miR-155 (red circle), miR-181b (green circle) and miR-21 (blue circle). The numbers in the intersections represent the number of target genes (390, 259 and 136) targeted by two miRs (miR-155 and miR-181b, miR-181b and miR-21 and miR-21 and miR-155, respectively) or by all of them (79 genes). The majority of genes (863 for miR-155, 1889 for miR-181b and 645 for miR-21) are targeted by only one miR

On the basis of the results on the experimental validation of gp120-modulated miRs in MDDCs, we integrated the initial target prediction analysis (Table 2) by using our previously published procedure [28] that exploits the predictions of three different algorithms (TargetScan, MiRanda and Pita; Additional file 3). By this *in-house* R bioconductor script we obtained the combined list of unique predictions for miR-21, miR-155 and miR-181b. The complete list of target genes for each of these three gp120-modulated miRs is reported in Additional file 4. We found that miR-21, miR-155 and miR-181b targeted 1119, 1468 and 2617 genes, respectively. To identify the genes targeted by more than one miR, we calculated the intersections among the three lists and represented them using Venn diagrams (Fig. 3). Interestingly, 79 genes were targeted by all miRs (Additional file 5). Information concerning the putative binding sites are provided in Additional file 6.

**Fig. 4:**
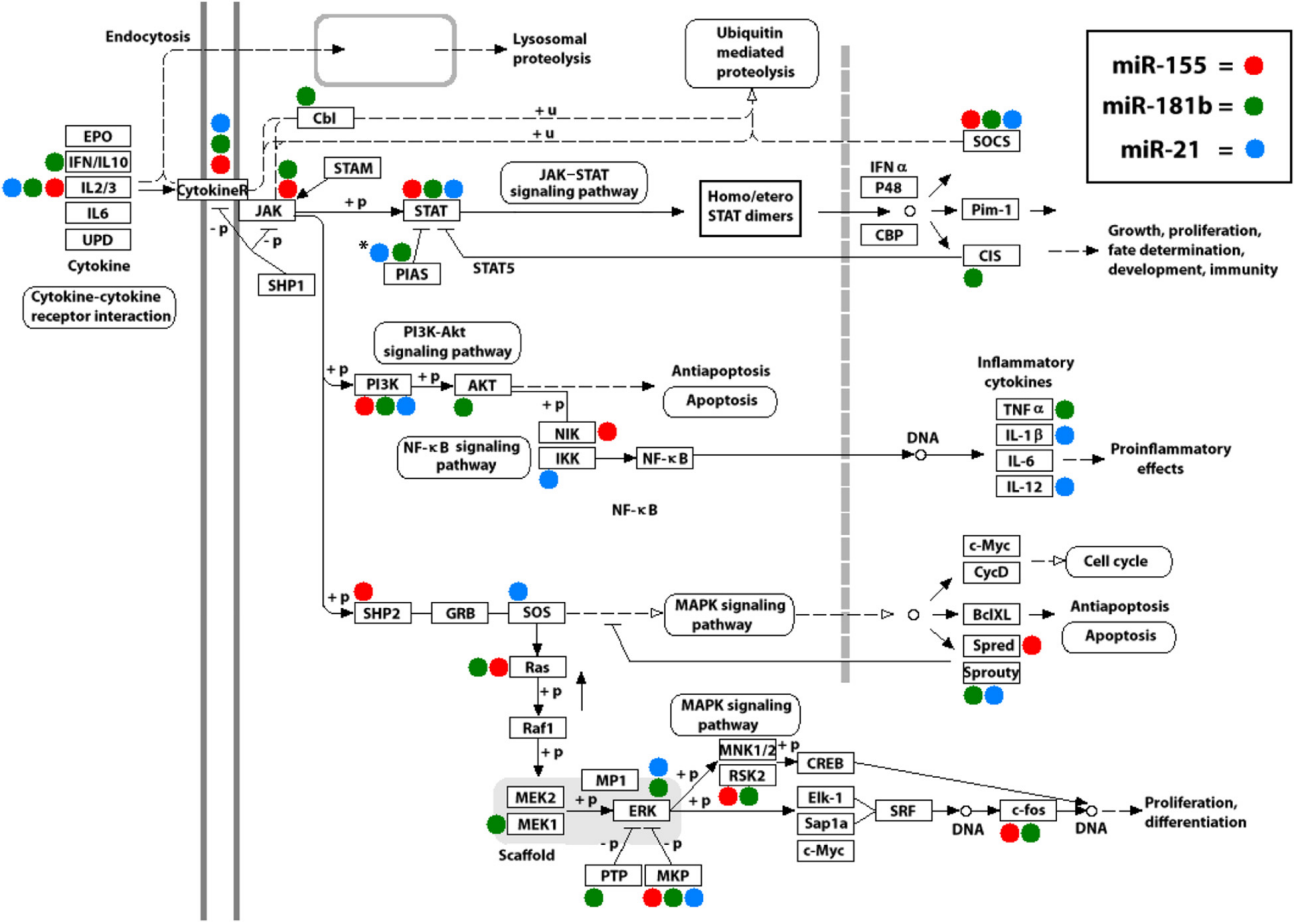
Interaction network of gp120-deregulated miRs and predicted targets in HIV gp120 exposed MDDCs. Many different pathways (JAK-STAT signaling, MAPK signaling, PI3k-Akt signaling, NF-kB signaling, apoptosis, and cytokine-cytokine receptor interaction) have been joined into a unified network of interactions, representing genes potentially involved in DC response to gp120. Three different colored dots were placed next to genes to indicate the putative targeting by miR-155 (red dot), miR-181b (green dot) or miR-21 (blue dot). *Not listed as predicted target but experimentally validated [65]

**Fig. 5:**
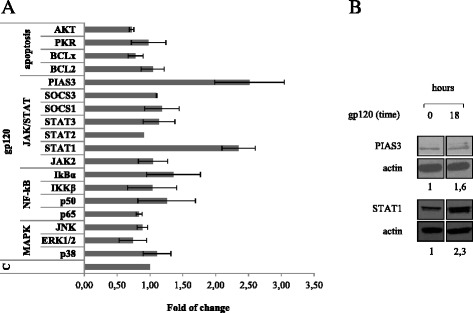
HIV gp120 up-regulates PIAS3 and STAT1 protein expression in MDDCs. MDDCs were stimulated with gp120 (5 μg/ml) for 18 h. Cell lysates were resolved by 8-12 % SDS-PAGE, transferred to a nitrocellulose membrane, and subjected to immunoblot analysis with antibodies specific for: p38, ERK1/2, JNK, p65, p50, IkBα, IKKβ, JAK2, STAT-1, −2, −3, SOCS-1, −3, PIAS3, Bcl2, BCLxL, Akt, and PKR. Data from one representative experiment are shown. **a** Graphs show the level of protein as determined by densitometry (ImageJ software) and calculated relatively to untreated control (**C**), where each sample was normalized to total actin. The average fold from two-four independent experiments was represented, along with the SD. **b** Immunoblot analysis with antibodies specific for PIAS3 and STAT1. Actin expression is shown as gel loading control. Values below the lanes show band intensities of the respective bands, normalized to actin expression. Data from one representative experiment out of four analyzed are shown

MiRs can regulate many target genes and modulate multiple pathways at the same time. To determine the biological processes and signaling pathways implicated in gp120-induced effects in MDDCs, the predicted targets of the newly identified gp120 deregulated miRs were separately subjected to gene ontology and KEGG pathway enrichment analysis, and annotated by means of DAVID bioinformatics tool [29]. Remarkably, the most significant (*p* < 0.05) categories of biological processes identified for miR-155 and miR-181b were transcription, regulation of transcription, regulation of RNA metabolic process, DNA-dependent regulation of transcription (Table 4). No statistically significant biological processes resulted for miR-21 targets, most probably due to the highly stringent selection criteria of the algorithm used (see additional file 3). By taking the combined list of unique predictions for miR-21, miR-155 and miR-181b, the bioinformatics analysis outlined the term ‘regulation of transcription’ as the biological process with the highest number of annotated targets (772 genes) (Table 4). As expected, many transcripts annotated in the JAK/STAT signaling pathway, including STAT members (STAT1 and STAT3), STAT regulatory factors (PIAS3 and SOCS), MAPK family members such as MAPK1 (ERK2), MAP2K1 (MEK1), MAP2K4 (MEK4), MAP3K1 (MEKK1), apoptosis mediators as well as cytokines and cytokine receptors (e.g. IL12A, IFNγ, CCR5, IL-1β) were found in this biological process. Furthermore, many of these genes belonged to more than one pathway, and were targeted by more than one miRs. Fig. 4 shows the network of interactions, which may be deregulated upon exposure of MDDCs to gp120 as a consequence of the altered expression of target genes, resulting from deregulation of miR-21, miR-155 and miR-181b. This network included cell growth, proliferation, fate determination, and development, immunity, pro-inflammatory effects and apoptosis pathways. As shown in Fig. 4, bioinformatics prediction outlined the presence of a huge number of potential targets for the three miRs, whose functional validation (i.e., protein expression) in our experimental model would require demanding and expensive analysis. Thus, to overcome these issues, we refined data by selecting a panel of genes targeted by one or more gp120 deregulated miR belonging to the signaling cascade previously reported to be altered in gp120-exposed MDDCs [14, 30, 31]. Among various targets, we analyzed the expression level of genes belonging to the JAK/STAT signaling pathway (i.e. STAT1, STAT2, STAT3, JAK2, PIAS3, SOCS1, SOCS3), MAPK/NF-kB signaling pathway (i.e. p38, ERK, JNK, p50, p65, IkBα, IKKβ), and apoptosis (AKT, BcL2, BCLxL, PKR), by western blotting analysis, in gp120-exposed MDDCs. As shown in Fig. 5, the results of protein expression analysis already reported in our previous study (STAT1, STAT2, STAT3, PIAS3, SOCS3, p38, p65, IkBα) or de novo carried out on the basis of bioinformatics analysis (ERK, JNK, p50, IKKβ, JAK2, SOCS1, AKT, BcL2, BCLxL, PKR) clearly indicate that among the putative targets analyzed, only PIAS3 and STAT1 protein expression levels are modulated, suggesting the involvement of gp120-deregulated miRs.

**Table 4:** Gene Ontology analysis for STAT3-deregulated miRNA (combined and individual predictions)

Gene Ontology ID	Gene Ontology Term	No. of genes	%	p-value	Corrected p-value (FDR)
*Target genes for miR-155*					
GO:0006350	transcription	246	16.8	1.06E-11	1.93E-08
GO:0045449	regulation of transcription	288	19.7	6.23E-11	1.13E-07
GO:0051252	regulation of RNA metabolic process	201	13.8	1.30E-07	2.35E-04
GO:0006355	regulation of transcription, DNA-dependent	197	13.5	1.57E-07	2.85E-04
*Target genes for miR-181b*					
GO:0045449	regulation of transcription	488	18.7	7.49E-13	1.40E-09
GO:0006350	transcription	406	15.6	2.00E-12	3.75E-09
GO:0051252	regulation of RNA metabolic process	345	13.2	1.05E-09	1.97E-06
GO:0006355	regulation of transcription, DNA-dependent	338	13.0	1.32E-09	2.47E-06
*Combined target genes for miR-21, miR-155 and miR-181b*					
GO:0006350	transcription	647	15.3	1.13E-19	2.15E-16
GO:0045449	regulation of transcription	772	18.2	4.94E-19	9.40E-16
GO:0051252	regulation of RNA metabolic process	539	12.7	9.54E-13	1.82E-09
GO:0006355	regulation of transcription, DNA-dependent	527	12.4	1.92E-12	3.66E-09
GO:0016481	negative regulation of transcription	154	3.6	1.88E-07	3.57E-04
GO:0010629	negative regulation of gene expression	166	3.9	2.28E-07	4.34E-04
GO:0045934	negative regulation of nucleobase, nucleoside, nucleotide and nucleic acid metabolic process	163	3.8	3.00E-06	5.72E-3
GO:0051172	negative regulation of nitrogen compound metabolic process	164	3.9	4.54E-06	8.64E-3
GO:0031327	negative regulation of cellular biosynthetic process	175	4.1	5.03E-06	9.57E-3
GO:0010558	negative regulation of macromolecule biosynthetic process	171	4.0	5.71E-06	1.09E-2
GO:0009890	negative regulation of biosynthetic process	176	4.2	1.29E-05	2.45E-2
GO:0000122	negative regulation of transcription from RNA polymerase II promoter	92	2.2	1.81E-05	3.45E-2
GO:0007167	enzyme linked receptor protein signaling pathway	113	2.7	1.97E-05	3.75E-2
GO:0006357	regulation of transcription from RNA polymerase II promoter	215	5.1	2.18E-05	4.16E-2

The table shows the Gene Onthology terms obtained after the bioinformatics analysis on the genes targeted by STAT3-regulated miRs, taken individually or in combination

## Discussion

miRs play important roles in many biological processes, including cellular defense against viral infection [32]. Increasing evidence has emerged that host miRs serve in animal cells to restrict viral infections. In turn, many viruses evolved strategies to counteract miRs action and may encode their own miRs [33]. In HIV-1 infection, many mechanisms underlying the capacity of the virus to induce immune destruction have been elucidated. However, the important role that miRs play in HIV-1 pathogenesis is only now emerging [9].

Studies carried out in PBMC highlighted the importance of the RNA interference machinery in HIV infection, in regulating viral replication [34] and latency [35, 36] as well as in defining different stages of disease [37]. Likewise, in monocytes/macrophages the differential expression of some miRs has been associated with susceptibility to infection [38]. Conversely, few studies have so far addressed the question of whether HIV-1 products may play a role in miRs modulation in the absence of productive infection. In this respect, Orecchini and co-workers reported a direct involvement of Tat, ectopically expressed, in the up-modulation of miR-222 [39]. Likewise, miR expression analysis carried out by Bignami and colleagues in healthy CD4+ T cells exposed to gp120 *in vitro*, outlined that miRs profile could be not only the result of a productive infection but also of exposure to HIV products that leave a signature in immune cells [37].

In keeping with these observations, and based on the identification of STAT3 binding motifs in the promoter region of a panel of selected miRs, we experimentally validated that MDDCs treated with gp120, show a down-regulation of miR-21 and miR-155 expression, whereas miR-181b is up-regulated at late time points. Interestingly, CCR5 engagement by one of its most specific ligand (i.e., CCL4) does not induce any change in the expression of these miRs, adding further evidence that the interaction of HIV-1 gp120 with CCR5 evokes complex and distinct signaling responses, differing from those activated by the coreceptors’ chemokine ligands [14, 40–46]. A growing body of evidence demonstrates that STAT3 signaling pathway is closely associated with the transcriptional regulation of some miRs including miR-21 and miR-181b [19, 47] as well as miR-155 [16, 18]. In keeping with these observations, we showed a striking direct relationship between STAT3 activation and expression of miR-21 and miR-181b since the gp120-induced deregulation of both miRs is completely reverted in the presence of Stattic. The time-course observed for the gp120-induced miR modulation support the hypothesis that late STAT3 activation via IL-6 production is responsible for this event as at earlier time points (6 h) we did not observe any changes in miRs expression. Several studies independently support the hypothesis of regulatory circuits between miR and STAT3 pathway in different cancer contexts linked to inflammation as a key component favoring tumorigenesis [15]. Of note, the miR we found concomitantly deregulated in DC upon exposure to gp120 have been previously reported to belong to a regulatory loop contributing to STAT3-mediated cancer development [15].

To the best of our knowledge, this is the first demonstration of HIV-induced miR modulation in human MDDCs. miRs are important regulators of DC differentiation and activation [27] and increased expression of miR-155 and miR-21 in DCs represents a general features associated with cell activation [24, 48]. Our finding that miR-21 and miR-155 are downregulated by gp120 together with our previous report that gp120 induces a tolerogenic-like DC phenotype [49] suggests that these HIV-modulated miRs could play a role in viral-induced DC dysfunction. In this regards, independent studies correlated miR-155 expression with HIV infectivity and spreading. In particular, Martinez-Nunez and colleagues demonstrated that increased expression of miR-155 correlates with reduced levels of DC-SIGN expression thus limiting gp120 binding to DCs [50]. Likewise, Napuri and co-workers highlighted a synergy between HIV-1 and cocaine to lower miR-155 and miR-20a in MDDCs that modulates DC-SIGN expression, DC maturation, and HIV infectivity [51]. Finally, silencing of miR-155 in murine DCs is associated with reduced production of IL-12 [52].

Moreover, gp120-deregulated miRs in MDDCs have been found to be concomitantly regulated in different experimental settings [53, 54], and reported to regulate important biological processes relevant to DC-HIV interaction, including NF-kB signaling pathway [19, 55, 56], DC maturation [23, 24, 48, 50], inflammation [56–58] and HIV replication [53]. By using a bioinformatics pipeline intersecting the predictions of three different algorithms [28], we identified ‘regulation of transcription’ as one of the most significantly enriched biological process for these miRs. Interestingly, among the overall list of miR targets, we found many genes related to the JAK/STAT signaling pathway, STAT regulatory factors, NF-kB/MAPK family members among others (Fig. 4). Several potential targets have been experimentally validated by assessing protein expression in MDDCs exposed to gp120. However, only two genes, that we previously reported to be regulated by gp120, i.e. STAT1 and PIAS3 were found to be modulated at the protein level. Although the observation that STAT3 activation is maintained despite the persistent PIAS3 up-regulation may at first glance appear contradictory, previous studies reported that STAT3 activation and high levels of inhibitory molecules co-exist up to several hours following stimulation [59]. Overall, this suggests that despite PIAS3 overexpression, its inhibitory function on STAT3 activation might be altered in the presence of gp120 thus contributing to chronic immune activation. Furthermore, it is also worth to be considered that although STAT3 protein levels are not modulated by gp120, the levels of STAT1 are increased at late time points. In this regard, it has been reported that STAT1 and STAT3 balanced expression or phosphorylation levels may somehow regulate the extent of inflammation [60].

## Conclusions

**Fig. 6:**
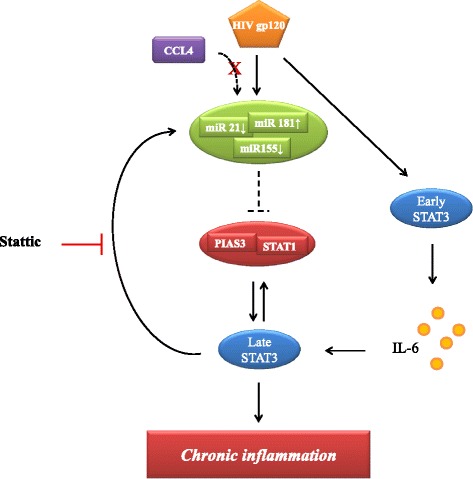
Schematic model of gp120-induced signalling pathways in MDDCs. The cascade of signals triggered by R5 HIV-1 gp120 in MDDCs is schematically shown

The new data provided by the present study add further complexity to the model we previously postulated to describe the interaction between DCs and HIV-1 in the absence of productive infection. As shown in Fig. 6, gp120 triggers a cascade of signaling events initiated by early activation of STAT3 leading to IL-6 production. Subsequently, IL-6 determines a second round of STAT3 activation responsible for miR regulation. Although it remains to be demonstrated that miR deregulation directly impact the function of molecular components belonging to STAT3 signaling, we exclude that these miRs directly regulate STAT3 expression as identical levels of protein are found independently of gp120 exposure of DC. Conversely, our data suggests that miRs could indirectly act on key regulators of STAT3/IL-6 axis ultimately contributing to the chronic immune activation observed in AIDS.

Overall, this study provides new evidence for the molecular mechanisms and signaling pathways triggered by HIV-1 in DCs, emphasizing the role of network interactions rather than individual connections among signaling components, transcription factors, miRs and their predicted targets in this process. Due to the complexity of these regulatory networks, large scale studies and comprehensive analyses are therefore needed to validate the role of these miRs in HIV infection, and determine the effect of their selective targeting in disease phenotype and outcome. Taken together experimental and bioinformatics analyses support the hypothesis that the STAT3-regulated miRs validated in our study may have a role in the DC response to HIV. In this scenario, the determination of STAT3-dependent miR deregulation, their hypothetical targets and interaction network may provide a broader view of the host-virus interactions.

## Methods

### Ethics statements

Healthy donor buffy coats were obtained from Centro Trasfusionale University of Rome’Sapienza”. Buffy coats were not obtained specifically for this study. Informed consent has not been asked because data were analyzed anonymously. Data from healthy donors have been treated by Centro Trasfusionale according to the Italian law on personal data management “Codice in materia di protezione dei dati personali” (Testo unico D.L. June 30, 2003 n. 196).

### Reagents

All culture reagents were purchased from Biowhittaker as endotoxin-free lots. Ultra pure LPS from *E. Coli* (serotype EH100, Ra TLRgrade) was purchased from Alexis Biochemicals (Nottingham, UK). Recombinant gp120 from CN54 HIV-1 strain was obtained from the national AIDS Research and Reference Reagent Program (Bethesda, MD). Recombinant CCL4 was purchased from R&D Systems. To test the effect of Stattic (Sigma, St Louis, MO), cells were treated prior to and during gp120 exposure with the inhibitor. Stattic did not exhibit any toxicity at the used concentration, as assessed by MTT assay (data not shown).

### Cell separation and culture

Monocytes were isolated from PBMCs obtained from healthy donor buffy coats by immunomagnetic selection using CD14 microbeads (MACS monocyte isolation kit from MiltenyiBiotec, Auburn, CA), according to the manufacturer’s instructions. This procedure yields a pure (≥98 %) population of monocytes, as assessed by FACS analysis of lineage specific surface markers (CD1a, CD14, CD3, CD19, CD56). To obtain immature MDDCs, monocytes were cultured at 1 x 10^6^ cells/ml in RPMI 1640 medium (Life Technologies, Gaithersburg, MD) containing 10 % FBS in the presence of GM-CSF (50 ng/ml) and IL-4 (500 U/ml). Cytokines were added to the cultures every 3 days. Both cytokines were kindly provided by Schering-Plough (Dardilly, France). On day 6, MDDCs were stimulated with LPS (10 ng/ml), gp120 (5 μg/ml), or CCL4 (100nM).

### Prediction of transcription factor binding sites

To predict the putative STAT3 binding sites on precursor region upstream of the miRNA sequence, we considered the list of miRNAs predicted to have putative STAT3 binding sites which has been recently reported [16, 17]. Since for most of these miRNAs the putative binding region spanned several kilobases from the transcription start site, the calculated binding score ranged from 100 to 1000 and most of them have a quite low STAT3 binding score (<500), we decided to refine the analysis by considering only those miRNAs with a higher STAT3 binding score (>500) and by limiting the prediction to a region of 3000 bases upstream the miRNA sequence. For these miRNAs, the upstream sequences were extracted by the University of California Santa Cruz genome browser (UCSC) (https://genome.ucsc.edu/). These sequences were then used to identify the STAT3 binding motif using the LASAGNA-search software (http://biogrid-head.engr.uconn.edu/lasagna_search/) as previously described [61].

### miRNA targets and STAT3 signaling pathway

Validated interactions among miRNAs and target genes belonging to STAT3 signaling pathway (Biocarta) were obtained by the curated database miRWalk (http://www.umm.uni-heidelberg.de/apps/zmf/mirwalk/) as previously described [62].

### Gene ontology and pathways analysis

To determine the biological processes and signaling pathways in which the predicted targets of the deregulated miRs were involved, we performed Gene Ontology, KEGG pathway enrichment analysis and annotation by means of DAVID bioinformatics tool [29].

### Expression of miRNAs

The expression of miRNAs were assessed by quantitative PCR (qPCR). Total RNA was extracted with the Total RNA Purification Plus Kit (Norgen Biotek, Canada) and converted into cDNA by using specific stem-loop primers [63]. The endogenous control let-7a was chosen as the endogenous miR since it displayed more constant expression values among all treatments with respect to U6 (data not shown). Relative quantification was performed by using the comparative Ct method [64]. qPCR was performed on an ABI-Prism 7900 HT (Lifetechnologies, Foster City, CA) using the SensiMix dT master mix (Bioline, London, UK), according to the manufacturer’s instructions.

### Immunoblotting

MDDCs were stimulated with gp120 (5 μg/ml) for 18 h. Cell were lysed in RIPA buffer (150 mM NaCl, 50 mM Tris-Cl (pH 7.5), 1 % Nonidet P-40, 0.5 % sodium deoxycholate, and 0.1 % SDS) containing a cocktail of protease and phosphatase inhibitors and protein extracts were resolved by 8-12 % SDS-PAGE, transferred to a nitrocellulose membrane, and subjected to immunoblot analysis with antibodies specific for STAT-1, STAT −3, SOCS1, NF-kB p50, IkBα, IKKβ, p38, ERK1/2, JNK, Akt and PIAS3 (Cell Signaling Technology; catalog numbers: 9172, 9139, 3950, 3035, 9242, 2684, 9212, 9102, 9252, 9272, 9042), and SOCS3, JAK2, STAT-2, NF-kB p65, Bcl2, BCLxL and PKR (Santa Cruz; catalog numbers: sc51699, sc476, sc81334, sc509, sc634, sc634, sc707), and actin (BD Transduction Laboratories; catalog numbers: 612656). Levels proteins were quantified using ImageJ software (software developed by Wayne Rasband, National Institutes of Health, Bethesda, MD).

### Statistical analysis

Statistical comparison between various groups was performed by Student’s *t*-test or one way analysis of variance (ANOVA) with either least significant difference (LSD) or Bonferroni post hoc tests as appropriate, using the SPSS software (12.0.2). Comparisons were made between means from several experiments. Differences were considered significant when *p* values were < 0.05. Statistical significance is indicated with * for *p* < 0.05.

### Deposition of data

Not applicable

### Availability of supporting data

Other information is provided as supplementary files

## Additional files

Additional file 1: Complete list of predicted STAT3 binding sites for each STAT3-regulated miRs with a binding score > 500. Additional file 2: Complete list of validated miR targets annotated in the STAT3 signaling pathway (Biocarta) listed together with the correspondent miR. Additional file 3: Bioinformatics workflow for target prediction. Additional file 4: Complete list of genes targeted by the gp120-modulated miR-21, miR-155 and miR-181b taken individually. Additional file 5: List of the common 79 genes targeted by the STAT3-regulated miR-21, miR-155 and miR-181b. Additional file 6: List of putative binding sites of genes targeted by STAT3-regulated miR.

## Abbreviations

ANOVA: Analysis of variance
LSD: Least significant difference
miR: microRNA
DC: Dentritic cell
MDDC: Monocyte-derived dendritic cell
qPCR: Quantitative PCR
