# Supplementary material for: HIV-1 gp120 influences the expression of microRNAs in human monocyte-derived dendritic cells via STAT3 activation
Source: BMC Genomics. 2015 Jun 27;16(1):480. doi: 10.1186/s12864-015-1673-3 (PMC4483217; doi:10.1186/s12864-015-1673-3)
Supplement: Additional file 3: — Bioinformatics workflow for target prediction. [file 12864_2015_1673_MOESM3_ESM.pptx]

## Slide 1
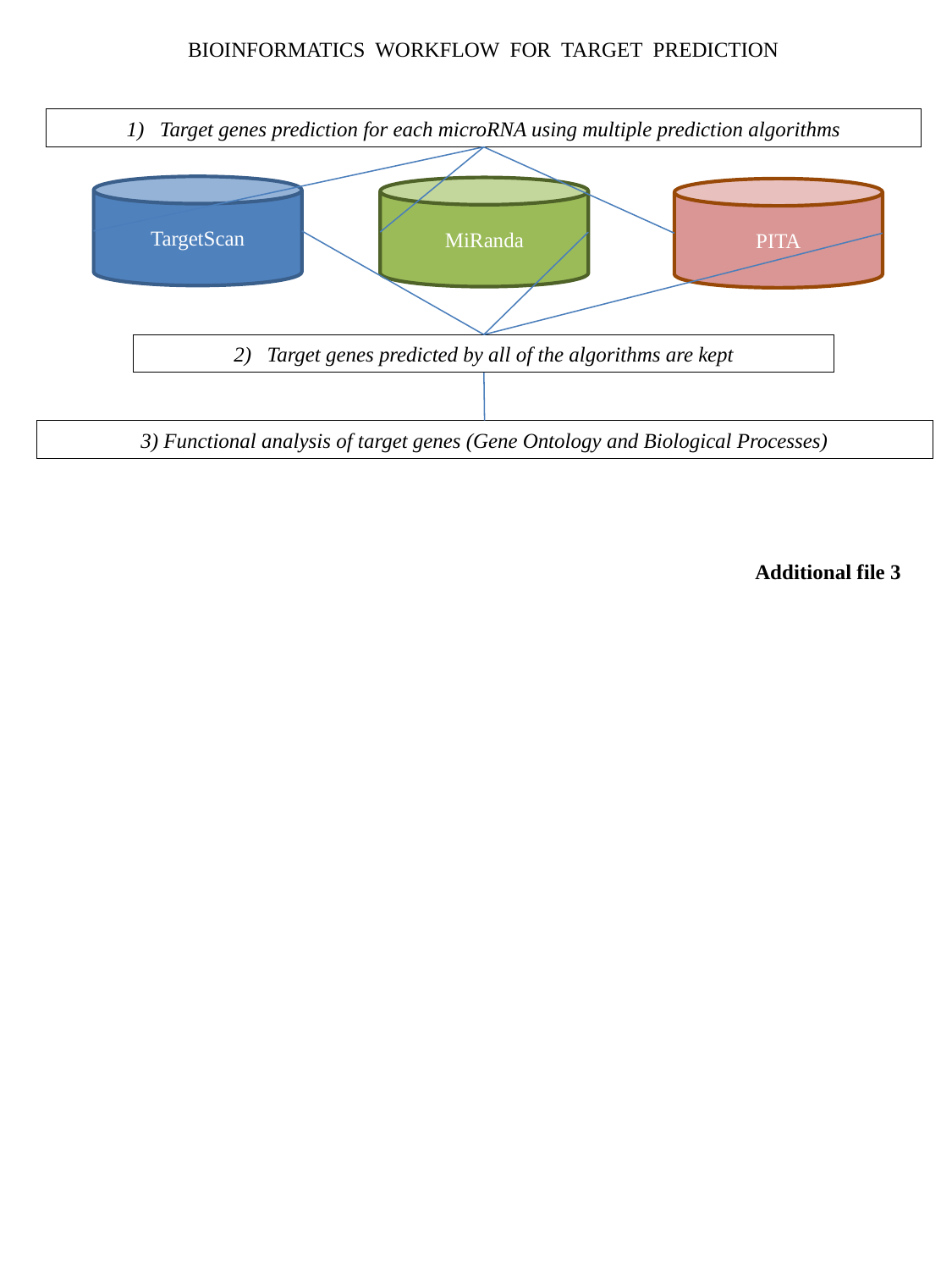

BIOINFORMATICS WORKFLOW FOR TARGET PREDICTION
1) Target genes prediction for each microRNA using multiple prediction algorithms
TargetScan
MiRanda
PITA
2) Target genes predicted by all of the algorithms are kept
3) Functional analysis of target genes (Gene Ontology and Biological Processes)
Additional file 3
